# Role of Dynamic Magnetic Resonance Imaging in Hirayama Disease, a Rare Motor Neuron Disease

**DOI:** 10.7759/cureus.31099

**Published:** 2022-11-04

**Authors:** Bhargavi Paladi, Dhanush Amin, Jyotsna Yarlagadda, Sriram Jadav, S. Afshan Jabeen

**Affiliations:** 1 Department of Radiology and Imageology, Nizam's Institute of Medical Sciences, Hyderabad, IND; 2 Department of Neurology, Nizam's Institute of Medical Sciences, Hyderabad, IND

**Keywords:** cervical spine, flexion mri, dynamic mri, laminodural space, hirayama disease

## Abstract

Introduction

Hirayama disease (HD) is a benign self-limiting motor neuron disease, most commonly occurring in young males. The disease has an insidious onset that affects T1, C8, and C7 myotomes. HD is more common in Japan and Asian countries, and rare in the western population. Magnetic resonance imaging (MRI) is the best technique for the diagnosis of this entity. Early diagnosis is important as the patients can be advised to limit neck flexion movements to arrest the progression of the disease. Any clinically suspected case of Hirayama disease should undergo flexion MRI as conventional neutral MRI may miss findings in a few cases. The purpose of the present study is to evaluate the usefulness of flexion MR imaging and laminodural space (LDS) measurement in young patients with clinical and electroneuromyography (ENMG) definite Hirayama disease.

Materials and methods

This is a retrospective observational study of 15 patients with clinical and ENMG definite Hirayama disease who were referred to the Department of Radiology. These patients underwent MRI of cervical spine in neutral position and with neck flexion of 30°-40°. In neutral MRI, atrophy and T2-weighted hyperintensities in the cord were noted. In flexion MRI, the maximum forward shifting of the posterior dural sac, also known as the LDS, was noted along with other parameters.

Observation

The mean age of the study population was 21 ± 3.36 years. Out of 15 patients, 14 were males and one was female; 14 patients (93.3%) had an involvement of unilateral upper extremity while one patient (6.6%) had asymmetric bilateral involvement. Straightening of cervical spinal curvature and cord atrophy was seen in 14 (93.3%) and 12 (80%) patients, respectively, on neutral position MRI. Intramedullary cervical cord T2-weighted hyperintensities were noted in eight patients (53.3%). Loss of the dural attachment and forward shifting of the posterior dural sac with prominent posterior epidural space was noted in all patients (100%). At the maximum forward shift of cord, the LDS ranged from 3.1 to 7.0 mm, with a mean of 5.38 ± 1.13 mm. Epidural flow voids were noted in 86.6% of cases.

Conclusion

Flexion MRI plays a very important role in confirming the diagnosis of Hirayama disease in clinically suspected cases. Anterior displacement of posterior dura matter and widening of LDS is noted in all cases in our study. Even though findings like cord atrophy and T2 hyperintensities are seen in conventional neutral MRI, these findings are not seen in all cases. So flexion MRI increases diagnostic confidence by showing increased LDS, which is a characteristic finding in Hirayama disease.

## Introduction

Hirayama disease (HD) also known as juvenile spinal muscular atrophy was first described by Hirayama in 1959 [1]. The disease is more common in Japan and Asian countries, and rare in the western population [2]. It is a benign, self-limiting motor neuron disease occurring in young males. The disease is predominantly sporadic with the course of the disease being initially progressive, which eventually becomes static [3]. Hirayama et al. confirmed that the central white and grey matter and the ventral root of the anterior horn of the spinal cord are the primary locations of the major lesion in HD [4].

The disease has an insidious onset that affects T1, C8, and C7 myotomes [5]. It usually presents with weakness and atrophy of the forearm and hand muscles, which spares the brachioradialis muscle, giving rise to oblique amyotrophy appearance [6]. 

There are multiple theories proposing the etiology of HD. One of them is necrosis of anterior horn cells caused due to microcirculatory changes [6]. The disease is usually unilateral with few patients having asymmetric or symmetric bilateral involvement [7]. Magnetic resonance imaging (MRI) is the modality of choice in this condition as it plays a crucial role in its diagnosis.

The aim of the present study is to evaluate the usefulness of dynamic MRI and laminodural space (LDS) measurement in young patients with clinical and electroneuromyography (ENMG) definite HD.

## Materials and methods

This is a retrospective observational study of 15 patients with clinical and ENMG definite HD who were referred to the Department of Radiology, Nizam's Institute of Medical Sciences, Hyderabad, India, from 2019 to 2021. Scans were done in 3 Tesla MRI (Skyra/Siemens Medical Systems, Germany) over the span of three years (2019-21).

Study protocol

Images of the cervical spine were initially acquired in a supine neutral position. Sagittal T2-weighted fast spin echo (FSE) with a repetition time (TR) of 4500 milliseconds (ms) and time to echo (TE) of 104 ms; saggital T1-weighted FSE with TR of 600 ms and TE of 8.7 ms; sagittal short tau inversion recovery (STIR) with inversion time (TI) of 220 ms, TR of 4600 ms, and TE of 55 ms; axial T2-weighted with TR of 3260 and TE of 90 ms; axial T1-weighted FSE with TR of 622 ms and TE of 11 ms sequences with a slice thickness of 3mm were acquired. Flexion T2 weighted FSE MRI of the cervical spine was performed with neck flexion of 30°-40° after providing support behind the nape of the neck and on either side of the neck to make sure that the immobility of the neck is maintained.

Image analysis

MR images were analyzed for spinal curvature, presence of cord atrophy, and T2-weighted hyperintensities in the cord in neutral MRI. The maximum forward shifting of the posterior dural sac (LDS) was measured on flexion sagittal T2-weighted imaging in the midline and the presence or absence of epidural flow voids was noted. At the site of maximum forward shift, anteroposterior cord and spinal canal diameter were measured in neutral and flexion images.

Statistical analysis

Observations were tabulated and data were presented as percentage, mean, and standard deviation.

## Results

Out of 15 patients, 14 (93.3%) were males and one (6.6%) was female. Patient ages ranged from 17 to 27 years with a mean age of 21 ± 3.36 years. The most common age group involved in our study was 15-19 years (53%) followed by 20-24years (27%). Fourteen patients (93.3%) had involvement of unilateral upper extremity while one patient (6.6%) had asymmetric bilateral involvement. Wasting and weakness of hand muscles were seen in all 15 patients (100%), and forearm muscles were involved in eight patients (53%). Four patients (26%) had hyperaesthesia in the hand.

**Figure 1:**
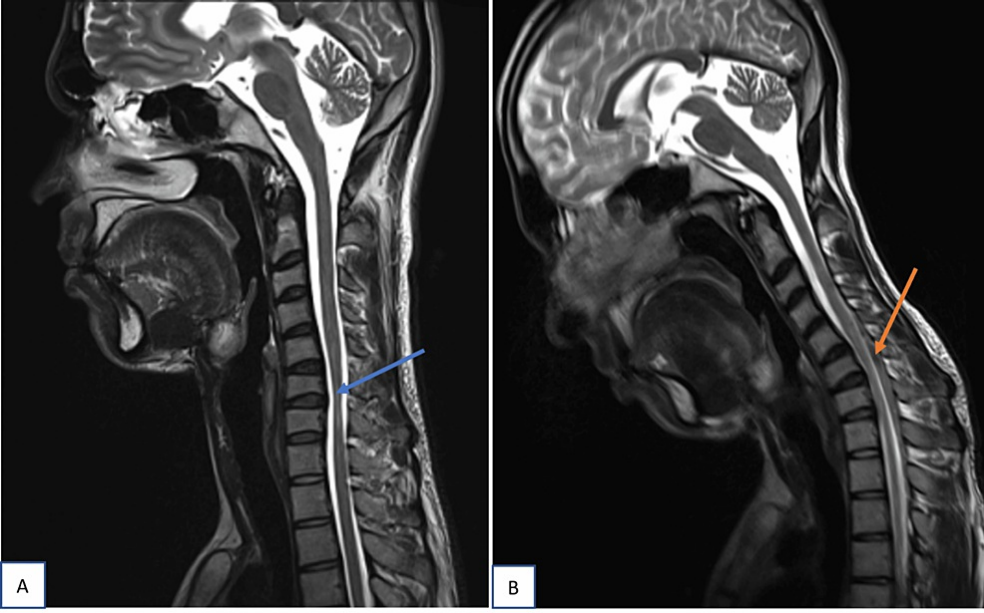
Sagittal T2W image of C spine in neutral and flexion position In neutral position (A), cord atrophy is noted at C6 to C7 level and T2 hyperintensities from C5  to C7 level (blue arrow). In flexion position (B), forward displacement of posterior dura (orange arrow)  from C5 to T1 level with maximum displacement at level of C6-C7 intervertebral disc. Laminodural space measures 5.2mm.

Neutral-position cervical MRI showed loss of cervical lordosis in 14 patients (93.3%) and localized lower cervical cord atrophy in 12 patients (80%). The cord atrophy was <2 vertebral heights in six patients (40%), 2-3 vertebral heights in five patients (33.3%), and >3 vertebral heights in one patient (6.7%). Intramedullary cervical cord T2-weighted hyperintensities were noted in eight patients (53.3%). Loss of the dural attachment, forward shifting of the posterior dural sac, and prominent posterior epidural space were noted in all 15 patients (100%) on flexion MRI (Figure 1). Flow voids in the posterior epidural space were noted in 13 patients (86.6%).

**Figure 2:**
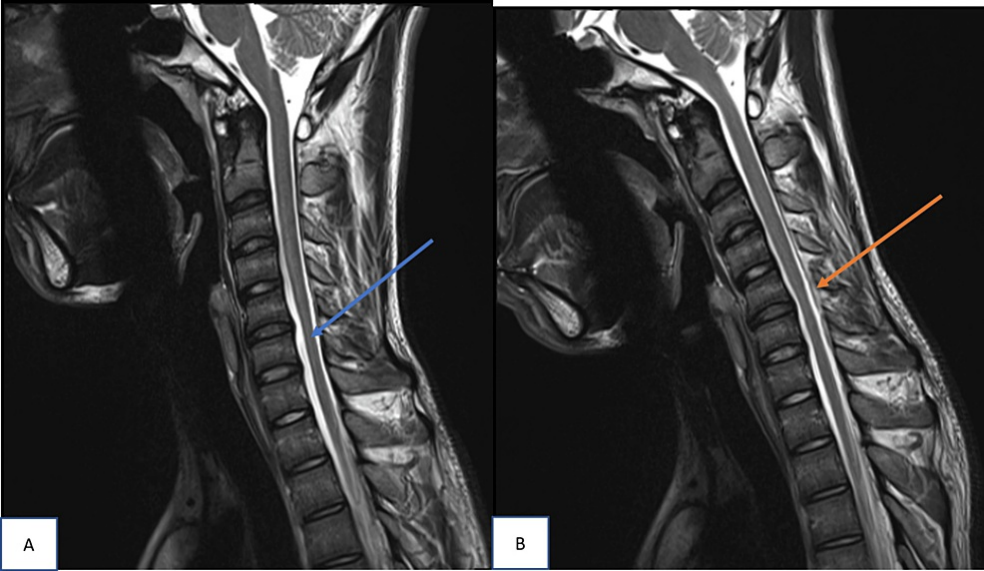
Sagittal T2W image of C spine in neutral and flexion position In neutral position (A), cord atrophy is noted at C5 to C6. No evidence of  T2 hyperintensities in the cord. In flexion position (B), forward displacement of posterior dura is noted from C4 to C7 level with maximum displacement at level of C5-C6  intervertebral disc. Laminodural space measures 3.1mm with no evidence of flow voids.

The LDS measurement varied from 3.1 to 7.0 mm, with a mean of 5.38 ± 1.13 mm (Table 1). Epidural flow voids were noted in 86.6% of cases (n=13). Two cases (13.3%) in the study did not show any epidural flow voids (Figure 2). These were cases with the least LDS measurements of 3.1 mm and 4.5 mm with lesser forward shifting of the posterior dural sac compared to others.

**Table 1:** Imaging parameters in neutral and flexion MRI in the current study LDS: laminodural space

	Minimum (mm)	Maximum (mm)	Mean	Standard Deviation
LDS Distance	3.1	7.0	5.38	1.136
Anteroposterior Diameter of Cord in Neutral MRI	3.3	7.1	4.66	0.772
Anteroposterior Diameter of Cord in Flexion MRI	3.4	6.8	4.166	0.94
Anteroposterior Diameter of Spinal Canal in Neutral MRI	8.3	11.5	9.713	0.86
Anteroposterior Diameter of Spinal Canal in Flexion MRI	4.2	10.5	6.32	1.522

The average canal diameter (anteroposterior) at maximum forward shifting was 6.3 ± 1.5 mm and 9.71 ± 0.86 mm during flexion and neutral MRI, respectively. The average cord diameter (anteroposterior) was 4.16 ± 0.94 mm and 4.66 ± 0.77 mm during flexion and neutral MRI, respectively.

## Discussion

HD is a self-limiting, benign, focal amyotrophy of the distal upper limbs. Early diagnosis is important as the patients can be advised to limit neck flexion movements to arrest the progression of the disease. For this, any clinically suspected case of Hirayama disease should undergo flexion MRI as conventional neutral MRI may not be positive in all cases.

It usually occurs in young men in the age group of 15 to 25 years [8]. In our study, 93.3% were males and most patients (53.3%) were in the age group of 15-19 years. Patients usually present with symptoms of unilateral upper extremity weakness and wasting, and cold paresis with no sensory or pyramidal involvement. In this study, the most common complaint was the insidious onset of weakness and wasting of hand muscles (100%) followed by weakness and wasting of forearm muscles (53.3%).

Though the asymmetric distribution of the disease is most common, there are case reports of a bilateral symmetric form of the disease [7]. C5-C7 segmental myotomes are commonly involved in this disease in the population of Western countries whereas C7-T1 segments are commonly involved in Asian countries [6]. In our study, C5-C6 levels were involved in all cases.

There are multiple theories proposed regarding the pathogenesis of Hirayama disease. Compression of the lower cervical cord by the posterior dural sac due to repeated or sustained flexion may result in chronic microcirculatory changes in the anterior spinal artery territory at the site of maximum kyphotic deformity leading to necrosis of the anterior horns of cord, which is the hallmark on pathology [5]. Imbalanced growth rates of the spine, spinal cord, and dural sac during the growth spurt might have caused a disproportion in the lengths of the vertebral column and the spinal canal contents. It may result in compression of the spinal cord by forward displacement of the posterior dural sac during neck flexion. At the end of the growth and development peak, because of the balance achieved among the above anatomical structures, patients gradually improve and the disease becomes self-limiting after two to four years of disease onset [9]. 

In healthy subjects, the dura is loosely suspended with several transverse folds, which compensates increased length of the cervical canal during flexion. Whereas, in Hirayama disease, a short dura cannot compensate for the increased length of the cervical canal during flexion and so the dura is displaced anteriorly resulting in compression of the spinal cord [10].

Shinomiya et al. suggested abnormal and unequal distribution of fine and larger ligaments between the posterior dura and ligamentum flavum may be the cause of asymmetric cord compression [11]. According to the theory by Ciceri et al, one of the causes of spinal cord ischemic changes might be venous congestion, which is secondary to impaired venous drainage occurring during flexion [12]. Upregulation of proinflammatory cytokines seen in cerebrospinal fluid of patients with Hirayama disease as proposed by Tanaka et al. might be one of the predisposing factors [13].

Routine radiographs of the cervical spine show straightening of spinal curvature, which is a nonspecific finding. Neutral position MRI imaging of the spine shows T2 hyperintensities in the cord and cord atrophy. However, these findings are not consistent. In a study done by Lehman et al., neutral MRI itself had high specificity and moderately high sensitivity for diagnosing Hirayama disease [14]. In our study, localized lower cervical cord atrophy was noted in 12 patients (80%). In a study conducted by Sonwalker et al. [15] and Raval et al. [16], cord atrophy was noted in 100% of cases. In our study, cord hyperintensities were seen in eight patients (53.3%). The proximal extent of cord atrophy and hyperintensities was C4 and the distal extent was T1 level.

**Figure 3:**
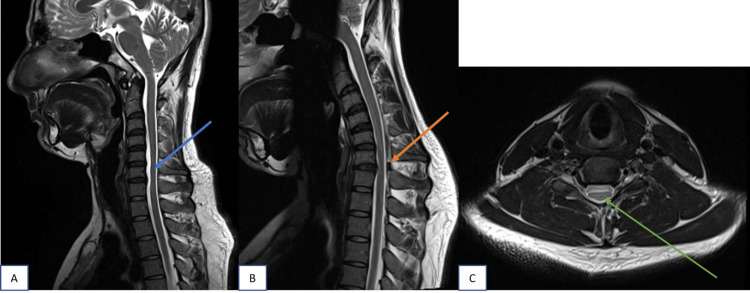
Sagittal T2W images of cervical spine in neutral and flexion position; axial image at the level of C6 vertebral body In neutral position (A), cord atrophy noted from C5 to C6 vertebral body level (blue arrow). No evidence of T2 hyperintensities in the cord . In flexion position (B), forward displacement of posterior dura (orange arrow) noted from C4 to C6 level with maximum displacement at level of C6 vertebral body. Axial T2W image (C) at level of C6 vertebral body showing asymmetrical flattening of cord (green arrow).

Cord atrophy and T2-weighted hyperintensities need not be present simultaneously in all cases. In the present study, there were five cases with cord atrophy but absent T2 hyperintensities, one case with cord T2 hyperintensities but no atrophy, and seven cases with both cord atrophy and T2 hyperintensities. There were two cases with absent cord atrophy and T2 hyperintensities but with increased LDS and epidural flow voids (Figure 3).

**Figure 4:**
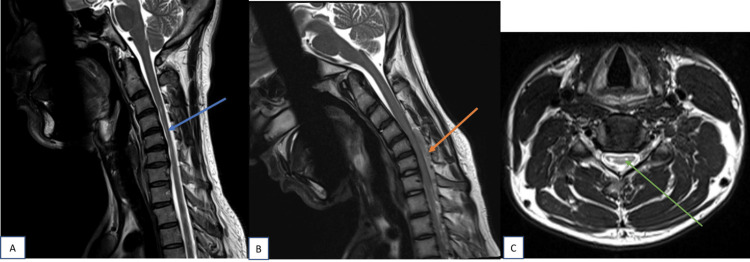
Sagittal T2W images of cervical spine in neutral and flexion position; axial image at the level of C5-C6 intervertebral disc In neutral position (A), cord atrophy and  T2 hyperintensities noted from C4  to C6 level (blue arrow). In flexion position (B), forward displacement of posterior dura (orange arrow) noted from C4 to T1 with maximum displacement at level of C5-C6 intervertebral disc. Axial T2-weighted image (C) at level of C5-C6 intervertebral disc showing asymmetrical flattening of cord and T2 hyperintensities with in giving owl-eyes (snake eyes) appearance (green arrow).

Asymmetrical cord flattening with central T2 hyperintensities in anterior horn cells giving classical owl-eyes sign (snake-eyes sign) can be seen on axial images (Figure 4).

**Figure 5:**
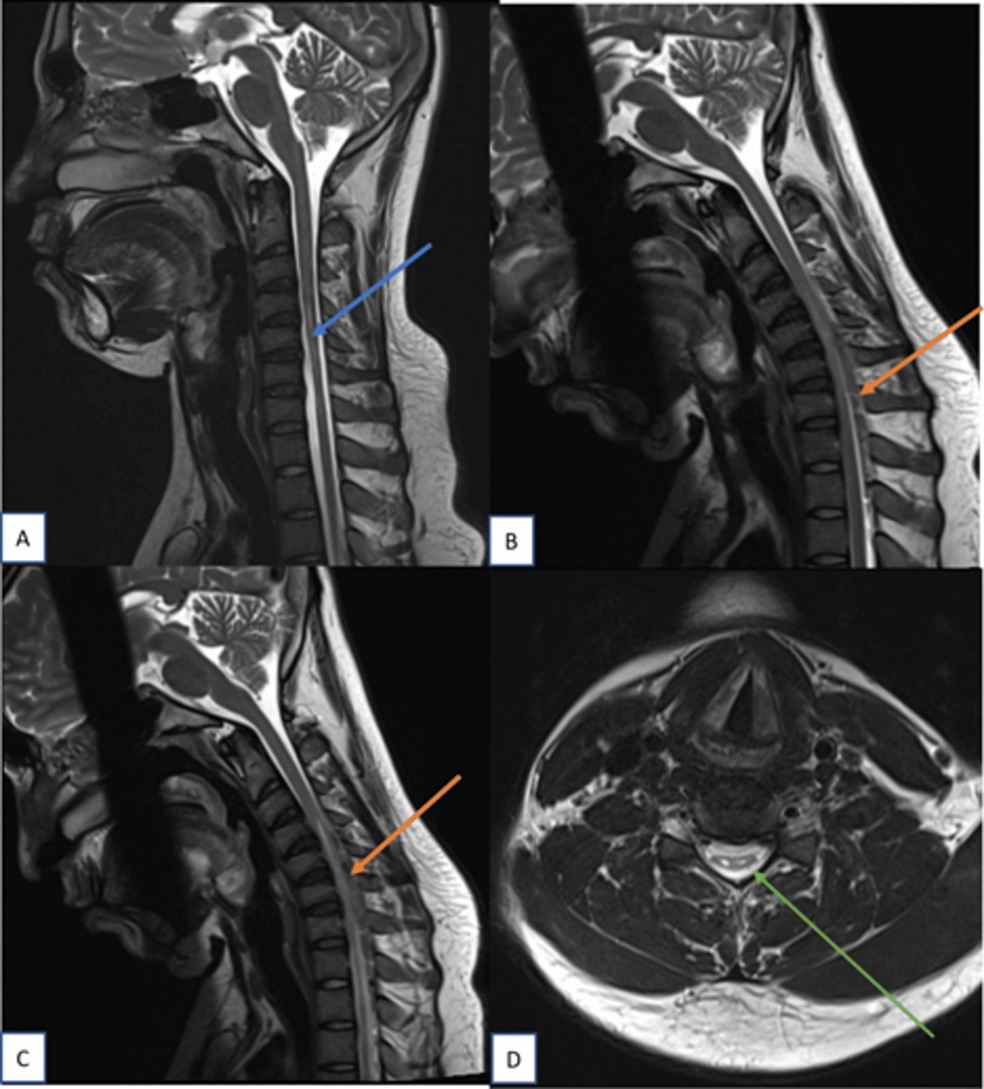
Sagittal T2W images of cervical spine in neutral and flexion position; axial image at the level of C6-C7 intervertebral disc. In neutral position (A), cord atrophy noted from C4  to T1  and T2 hyperintensities from C4  to C7 (blue arrow). In flexion position (B,C), forward displacement of posterior dura (orange arrow) noted from C4 to T3 with maximum displacement at level of C6-C7 intervertebral disc. Axial T2-weighted image (D) at level of C6-C7 intervertebral disc showing asymmetrical flattening of cord and T2 hyperintensities within (green arrow).

Flexion MRI spine shows forward displacement of the posterior dural sac with increased LDS and epidural flow voids. Flow voids within the epidural space can be visualized on conventional MRI though poorly in a few cases. Epidural flow voids were seen in 86.6% of cases in our study. According to Sonwalkar et al., dynamic post-contrast imaging is an important technique in workup of HD [15]. Fast imaging employing steady-state acquisition (FIESTA) sequence showed prominent flow voids within the epidural mass in all patients, in a study conducted by Raval et al. [16]. Epidural flow voids were better appreciated on three-dimensional constructive interference in steady state (3D-CISS) sequence, as suggested by Gupta et al. [17]. In our study, we used only routine non-contrast MRI sequences (Figure 5).

Flexion cervical MRI showed forward shifting of the posterior dural sac (increased LDS) in all 15 patients (100%). In a study by Zhou et al., forward shifting of the posterior dural sac was seen in 71% of the cases [18]. Lai et al. observed that an increase in LDS was also seen in up to 46% of healthy subjects, though without cord compression. They noted that the increase in LDS was mild in those healthy individuals, ranging from 1.0 to 4.2 mm with a mean of 1 mm, compared with those with HD in whom it ranged from 6.1 to 7.8 mm, with a mean of 6.7 mm [19]. In a study conducted by Boruah et al. on 45 patients, the mean LDS was 5.99 ± 1.9 mm [20]. In the current study, the increase in LDS ranged from 3.1 to 7.0 mm, with a mean of 5.38 ± 1.13 mm.

In our study, the average canal diameter (anteroposterior) at maximum forward shifting was 6.3 ± 1.5 mm and 9.71 ± 0.86 mm during flexion and neutral MRI, respectively. This finding indicates a decrement in the anteroposterior dimension of the spinal canal during flexion MRI. The average cord diameter (anteroposterior) was 4.16 ± 0.94 mm and 4.66 ± 0.77 mm during flexion and neutral MRI, respectively. The decrease in the anteroposterior diameter of the spinal cord is due to compression and anterior displacement of the cord by the posterior epidural component, which occurs during flexion.

## Conclusions

Flexion MRI plays a very important role in confirming the diagnosis of Hirayama disease in clinically suspected cases. Anterior displacement of posterior dura matter and widening of LDS was noted in all cases in our study. Even in conventional neutral MRI, findings like cord atrophy and T2 hyperintensities are seen but not in all cases. So flexion MRI increases diagnostic confidence by showing increased LDS, which is a characteristic finding in HD.
